# Initial Clinical Experience of Repeat Thrombectomy with a Retrieval Stent (RTRS) with Continuous Proximal Flow Arrest by Balloon Guide Catheter for Acute Intracranial Carotid Occlusion

**DOI:** 10.1155/2021/7607324

**Published:** 2021-12-31

**Authors:** Wen-huo Chen, Tingyu Yi, Yan-Min Wu, Zhi-nan Pan, Xiu-fen Zheng, Xiao-hui Lin, Ding-lai Lin, Rong-cheng Chen

**Affiliations:** Department of Neurointervention, Zhangzhou Affiliated Hospital of Fujian Medical University, Fujian, China

## Abstract

**Background:**

Balloon guide catheters (BGCs) have good performance in terms of radiological outcomes in acute ischemic thrombectomy. It is not uncommon for BGCs to be blocked by thrombi, especially in cases with acute intracranial internal carotid artery (ICA) occlusion. Our initial experience using repeat thrombectomy with a retrieval stent (RTRS) with continuous proximal flow arrest by BGC for acute intracranial ICA occlusion is presented.

**Methods:**

In patients with acute intracranial ICA occlusion treated with RTRS, clinical data, including the National Institutes of Health Stroke Scale (NIHSS) score at admission and modified Rankin Scale (mRS) score at 90 days, and procedural data, including the Extended treatment in Cerebral Infarction (eTICI) score, procedural time, and complications, were analyzed.

**Results:**

Thirty-two consecutive patients (12 men (37.5%); mean age: 73 years) were treated with RTRS using a BGC. The median NIHSS score was 19. The median puncture-to-reperfusion time was 46 minutes (range: 22-142 minutes). All patients were successfully revascularized; eTICI 2c or better recanalization was achieved in 30 (93.8%) patients. No procedure-related complications or symptomatic intracranial hemorrhage occurred. Two cases (6.3%) had distal emboli, but none had emboli to the anterior cerebral artery. Fourteen patients (43.8%) achieved a good outcome with an mRS score of 0–2 at 90 days, and 8 patients (25.0%) died.

**Conclusions:**

In patients with intracranial ICA occlusion, RTRS with proximal flow arrest by BGC is effective and safe, achieving good clinical and angiographic outcomes. This method may reduce the incidence of distal emboli in thrombectomy with stent retrievers.

## 1. Introduction

Acute intracranial internal carotid artery (ICA) occlusion, especially carotid T occlusion, is often associated with a large clot burden, low clot burden score, poor collateral flow, and poor outcome, resulting in a large ICA territory infarction [1]. Its overall mortality is up to 51% [1]. Endovascular therapy is the standard treatment for acute large intracranial artery occlusion [2–8]. Despite major strides in reducing disability from large vessel occlusion strokes with stent retrievers (SRs), 72% of patients with intracranial ICA occlusion remain physically disabled [1]. A high first-pass complete reperfusion rate [9], short puncture-to-reperfusion time (PRT) [10], and low incidence of distal emboli [11] are associated with improved functional neurological recovery.

Balloon guide catheters (BGCs) offer reliable blood flow arrest and flow reversal in combination with aspiration via syringes or high-flow pump systems [12]. Nonrandomized studies suggest that BGC use during mechanical thrombectomy for acute ischemic stroke is associated with superior clinical and angiographic outcomes [13]. However, in the setting of acute intracranial ICA occlusion with a large clot burden, it is very possible that BGCs is blocked by thrombi. In the clinical setting, when the guide catheter is blocked by a thrombus, no blood flow comes out of the catheter, and the guide catheter will be retracted to clear the thrombus in it. However, such an operation wastes time and may increase the possibility of distal emboli occurrence, which may influence patients' clinical outcome.

The purpose of our study was to assess the feasibility of repeat thrombectomy with a retrieval stent (RTRS) with continuous proximal flow arrest by BGC and to evaluate whether the use of this technique improves reperfusion and clinical outcomes in acute intracranial ICA occlusion.

## 2. Methods

### 2.1. Study Patients

The study included patients identified from our prospective registry database of acute stroke patients treated with endovascular therapy between January 1, 2015, and November 30, 2020. The selected patients met the following criteria: (1) ischemic stroke resulting from acute intracranial ICA occlusion confirmed by digital subtraction angiography (DSA); (2) patient age greater than 18 years; (3) prestroke modified Rankin Scale (mRS) score of 0–1; and (4) receiving endovascular therapy with the use of a BGC.

The exclusion criteria for this study were thrombectomies performed for (1) ipsilateral stenosis or acute extracranial occlusions, (2) previously deployed stents, and (3) dissections and in settings in which (4) the BGC was not blocked by a thrombus during the procedure and (5) the BGC was blocked by a thrombus but the RTRS technique was not used.

### 2.2. Endovascular Procedure: RTRS Technique

**Figure 1:**
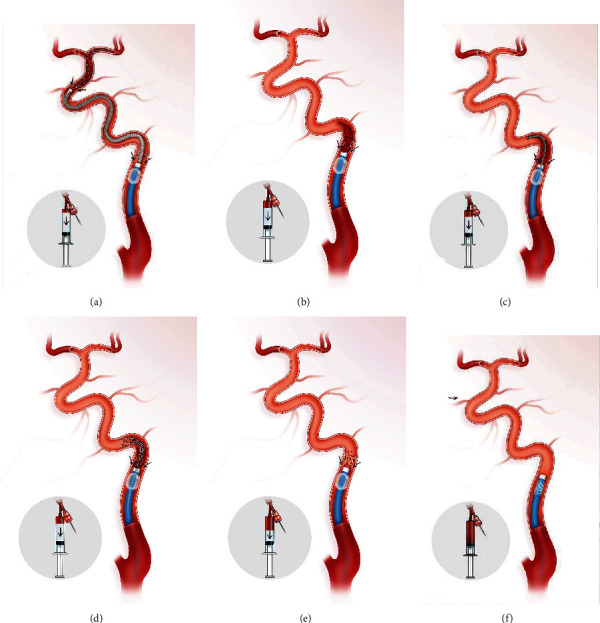
Illustration of the repeat thrombectomy with a retrieval stent (RTRS) technique with continuous proximal flow arrest by a balloon guide catheter for acute intracranial ICA occlusion. (a) DSA shows ICA terminus occlusion involving the MCA and ACA. The retrieval stent is unsheathed at the occlusion site. After at least 3 minutes, the balloon is inflated to arrest the anterograde flow from the ICA, and then the fully deployed Solitaire stent is partially resheathed. Together with the delivery microcatheter and distal access catheter, the stent is gently pulled back under continuous suction achieved with a syringe. This procedure is called primary clot retrieval. (b) The BGC was blocked by a thrombus, and there was no blood flow through the BGC. The BGC was kept inflated, and continuous suction was achieved via a syringe. (c) The microcatheter and stent were advanced together directly into the site, which was close to the distal tip of the BGC. (d) The stent is unsheathed at the site close to the distal tip of the BGC, and then the stent together with the delivery microcatheter is retrieved back under continuous suction. This procedure is called rescue clot retrieval. (e) Blood flow without a thrombus comes out of BGC, and gentle injection contrast via the BGC was performed to confirm successful reperfusion. (f) The BGC was deflated. ICA: internal carotid artery; DSA: digital subtraction angiography; BGC: balloon guide catheter.

The RTRS technique is shown in Figure 1.

All procedures were performed under general anesthesia or conscious sedation. After femoral puncture with an 8F sheath, 2500 units or less of intravenous (IV) heparin was injected. An 8F BGC (Merci/Flowgate 2, Stryker Products, Boston, USA) was advanced with a 4F/5F MPA catheter or via the exchange technique and placed distal to the carotid bifurcation. After the presence of acute intracranial ICA occlusion was confirmed by injection of contrast through the BGC and in some cases a distal access catheter (Catalyst, Stryker Products, Boston, USA; Navein, ev3 Inc., Plymouth, MN, USA) was applied, a 0.014-inch microwire (Transcend; Stryker, Kalamazoo, MI) was passed through the intracranial ICA occluded segment, and a microcatheter (ev3 Inc., Plymouth, MN, USA) was advanced over the microwire through the occlusion site. Microcatheter injection was performed to verify the microcatheter's position distal to the thrombus; then, a Solitaire AB 6-30 mm (ev3 Inc., Plymouth, MN, USA) was unsheathed at the occlusion site. After at least 3 minutes, the balloon was inflated to arrest the anterograde flow from the ICA, and the fully deployed Solitaire stent was partially resheathed by microcatheter or distal access catheter. Together with the delivery microcatheter and distal access catheter if used, the stent was gently pulled back under continuous suction achieved with a syringe (Figure 1(a), named as primary clot retrieve). If the BGC was blocked by a thrombus and there was no blood flow through the BGC, the BGC was kept inflated and continuous suction was applied via a syringe (Figure 1(b)). Then, the microcatheter together with stent was advanced directly into the site, which was close to the distal tip of the BGC (Figure 1(c)), and unsheathed at the site. The stent together with the delivery microcatheter was then retrieved under continuous suction (Figure 1(d), called rescue clot retrieval). Steps C and D were repeated (i.e., the repeat thrombectomy with a retrieval stent (RTRS) technique) with continuous inflation of the balloon until there was blood flow through the BGC without a thrombus (Figure 1(e)). Contrast was injected via the BGC to confirm successful reperfusion was achieved; then, the BGC was deflated (Figure 1(e)).

### 2.3. Outcome Measures

#### 2.3.1. Clinical Outcomes

The primary clinical efficacy outcome was the rate of good prognosis at 90 days postprocedure, as defined by an mRS score of 0 to 2. Safety was evaluated by the incidence of symptomatic intracranial hemorrhage (sICH) and all-cause mortality ≤ 90 days postprocedure.

#### 2.3.2. Angiographic and Procedural Outcomes

The primary angiographic outcome was the rate of achieving successful reperfusion (eTICI ≥ 2b, eTICI ≥ 2c, and eTICI 3), [14] after a single pass of primary clot retrieval. Achievement of complete reperfusion (eTICI 3) after a single pass of primary clot retrieval no matter how many passes of rescue clot retrieval was adpotted, is called the first-pass effect (FPE) [15].

Secondary outcome measures included the incidence of distal emboli, the incidence of emboli in the anterior cerebral artery (ACA), and the final successful reperfusion rate. Successful reperfusion was defined as an eTICI grade of 2b, 2c, or 3 after endovascular treatment. The PRT was defined as the time from puncture to achieving eTICI ≥ 2b or procedure completion.

### 2.4. Data Availability

Access to patient records for data collection and analysis was approved by our local medical ethics committee, and informed consent was not obtained because of the retrospective nature of the study. We will share the identified data of participants in our study upon request.

### 2.5. Statistical Analysis

The data for categorical variables are described in absolute and relative frequencies. The data for continuous variables are given as the median and range or the mean and standard deviation. All statistical analyses were performed with IBM SPSS Statistics 22.0 (IBM, Inc., Armonk, NY).

## 3. Results

**Figure 2:**
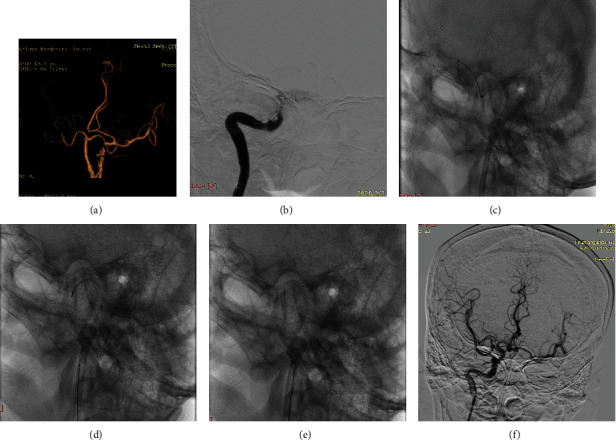
An elderly presented with left hemiparesis and a history of atrial fibrillation postcardiac valve replacement. The patient regularly took warfarin, and admission NIHSS was 18. (a) Computed tomography angiography (CTA) shows total occlusion of the right ICA occlusion and the anterior communicating artery open; the right anterior cerebral artery is supplied by the left ICA. (b) A Solitaire 6-30 mm stent was unsheathed at the occlusion site (shown as a black arrow). DSA showed ICA intracranial segment occlusion with a large clot burden, and the CBS was 3. (c) After at least 3 minutes, the balloon (shown as a black arrow) was inflated to arrest the anterograde flow from the ICA. Then, the fully deployed Solitaire stent was partially resheathed, and together with the delivery microcatheter, it was gently pulled back under continuous suction achieved with a syringe. This procedure is called primary clot retrieval. (d) The BGC remained inflated for there is no blood out of BGC, and continuous suction was applied via a syringe; the microcatheter and stent are advanced together directly into the site, which is close to the distal tip of the BGC. (e) The stent is unsheathed at the site close to the distal tip of the BGC, and then the stent together with the delivery microcatheter is retrieved back under continuous suction. This procedure is called rescue clot retrieval. (f) Rescue clot retrieval is repeated until there is blood flow without a thrombus through the BGC; contrast is gently injected via the BGC to confirm that successful reperfusion is achieved. The balloon is deflated, and DSA shows that the right ICA is totally patent and the anterior communicating artery is open; the bilateral MCA and ACA are supplied by the right ICA. NIHSS: NIH stroke scale; CTA: CT angiography; DSA: digital subtraction angiography; ICA: internal carotid artery; BGC: balloon guide catheter; CBS: clot burden score; MCA: middle cerebral artery; ACA: anterior cerebral artery.

From 1 January 2015 to 1 November 2020, there were 426 patients with acute intracranial ICA occlusion, and BGCs were used in 116 patients. The BGC was blocked by a thrombus after the first pass of SR, allowing no blood flow out of the BGC, in 40 patients (36 cases with 8F Merci, 4 cases with 8F Flowgate 2), and the RTRS technique was applied in 32 patients. An illustration of the cases is shown in Figure 2.

Baseline characteristics, clinical features, and preprocedural radiological features are shown in Table 1. There were 12 (12/32, 37.5%) male patients, the age of onset was 73 ± 11 years, and the mean admission NIHSS score was 19. Regarding vascular risk factors, 23/32 (71.9%) patients had hypertension, 6/32 (18.8%) had diabetes mellitus, 3/32 (9.4%) had hyperlipidemia, 21/32 (65.6%) had atrial fibrillation, and 7/32 (21.9%) were smokers.

Procedure details and radiological and clinical outcomes are shown in Table 2. Regarding procedural details, all patients underwent one pass of primary clot retrieval, the median number of rescue clot retrieval passes was 1 (range from 1to 8), the mean PRT was 61.2 ± 24.0 minutes, the median PRT was 46 minutes, and the range was from 22 minutes to 142 minutes. After one primary clot retrieval pass, eTICI ≥ 2b reperfusion was achieved in all patients, eTICI ≥ 2c reperfusion was achieved in 30/32 (93.8%) patients, eTICI 3 reperfusion was achieved in 27/32 (84.4%) patients, distal emboli occurred in 2/32 (6.3%) patients, and no cases of emboli to the anterior cerebral artery or sICH occurred.

Regarding clinical outcome, the good prognosis rate was 43.8% (14/32), and the mortality rate was 25.0% (8/32).

## 4. Discussion

RTRS is a modification of the conventional SR thrombectomy technique. The modifications of conventional SR thrombectomy are (1) the use of a BGC with the SR thrombectomy technique, (2) continuous inflation of the balloon when removing the retrieval stent and distal access catheter from the guide catheter, and, most importantly, (3) the inclusion of a procedure for when the BGC is blocked by a thrombus and no blood flows out of the BGC. In RTRS, the balloon is kept inflated, and then repeated thrombectomy with a retrieval stent is performed at the site near the distal tip of the BGC until blood flows out of the BGC. Angiography is performed via the BGC by gentle manual injection of contrast agent to ensure that the thrombus is cleared, and then, the balloon is deflated.

The use of RTRS when the BGC is blocked by a thrombus has three useful advantages. First, it is time saving. The BGC does not need to be retracted out of the sheath and reestablished the route. During the rescue clot retrieval procedure, we did not need to advance a microcatheter with a microwire into the middle cerebral artery. We just advanced the microcatheter together with the stent into the site that was close to the distal tip of the BGC and unsheathed the stent at that site, and such an operation was very quick, simple, and convenient. Our study showed that the mean time of PRT was shorter than that in a previous study, which was also about endovascular therapy for intracranial artery occlusion (59 minutes vs. 120 minutes) [1]. Second is the high rate of successful reperfusion. Acute intracranial ICA occlusion, especially carotid T occlusion, is usually associated with high clot burden and low CBS [16], and the successful reperfusion rate ranges from 60.5% [1] to 86.9% [17]. In our study, all patients achieved successful reperfusion, and approximately 90.9% of patients achieved nearly complete successful reperfusion after one primary clot retrieval pass. Third is high FPE occurrence. FPE is associated with significantly higher rates of good clinical outcome [9, 15]. A previous study showed that FPE was observed in 33% of patients with intracranial artery occlusion who underwent SR thrombectomy with 8F Flowgate 2BGC in which the inner lumen was larger than that of 8F Merci. In our study, FPE was observed in 81.8% of patients. Fourth, there was a low occurrence of distal emboli and emboli into the ACA. Distal emboli are a troublesome event during mechanical thrombectomy and is usually associated with increased mortality and disability rates, regardless of the success of reperfusion [18]. Distal emboli are a controllable event. Acute intracranial ICA occlusion, which is associated with a high clot burden, had a higher rate of distal emboli (15.3%) than middle cerebral artery (MCA) occlusion (4.8%) [18]. The use of mechanical thrombectomy (MT) techniques can decrease the incidence of distal emboli. For example, the use of distal access catheters in association with SR, which is common in clinical practice, can decrease the distal emboli incidence from 5% to 11% [19]. The use of BGCs can also decrease the incidence of distal emboli [20, 21]. Regarding intracranial ICA occlusion, the incidence of distal emboli is as high as 75% in patients who underwent MT with conventional guide catheters [20], but the incidence decreases to 0% when MT is combined with BGCs [20]. In our study, the incidence of distal emboli was 6.3%, with no emboli to the ACA. Regarding cases with distal emboli, the thrombus is very small, so it causes only very remote branches to become occluded, which may minimize the influence on functional outcome.

There are three important technical points of the RTRS technique. The first point is continuous inflation of the balloon when there is no blood flow coming out of the BGC and never performing angiography with contrast during this step because of the risk of moving the thrombus into a distal territory with the injection pressure. Second, repeat thrombectomy with a retrieval stent should not be stopped at the site near the tip of the BGC until blood is flowing out of the BGC and no thrombus is trapped by the stent. Third, when the blood flow from the BGC is not fluent and no thrombus is trapped by the stent, two situations should be considered. One is that the tip of the BGC is completely or partially covered by the vessel wall, and the other is that there is a thrombus in the ICA terminus. Therefore, we needed to partly unsheath the stent at the ICA terminus, deflate the balloon, gently retract the BGC slightly, and then perform angiography via the BGC to check what happened. If complete recanalization is achieved, the procedures terminated; if there is thrombus in the culprit artery, the balloon is reinflated, and the thrombectomy was repeated.

There is one concern about cerebral ischemia caused by continuous proximal blood flow arrest using balloon occlusion, which has also been reported with the TSAT technique [21]. However, we only adopt this technique in patients with intracranial ICA occlusion, so we do not need to worry about this problem because the vessel is initially occluded and there is no blood supply downstream territory. Another concern is the formation of fresh thrombi secondary to continuous proximal blood flow arrest. Therefore, the procedure should be performed as soon as possible, and proper heparinization is also needed during the procedure.

Due to the excellent performance of the RTRS technique on radiological outcomes, the clinical outcome of the patients in our study was acceptable and even better than that of those in a previous study, although the thrombus burden was large in our included cases, which is usually associated with poor clinical outcomes. The clinical outcome is poor in patients with intracranial ICA occlusion [17, 22, 23], the rate of good outcomes ranges from 21.4% [1] to 40% [17], and the mortality rate ranges from 29.3% [22] to 55.2% [1]. In our study, the incidence of good outcomes was 43.8%, and the mortality rate was 25.0%.

This study is limited by being a single-center study. Despite this limitation, our study is the first series with relatively numerous cases that attempted to examine an endovascular strategy in the setting of the BGC being blocked by a thrombus. Second, the 8F Flowgate2 BGC inner lumen (0.084 inches) of which is larger than that of the 8F Merci (0.078 inches), was not used in all cases in our study. However, we only present the technique to address the circumstance of the BGC being blocked by a thrombus, which could also occurred in cases with 8F Flowgate 2 BGCs or even those with larger inner lumens (0.090 inches) [24], so this usage of this technique is not influenced.

## 5. Conclusion

RTRS with proximal flow arrest using a BGC is effective for the treatment of acute ischemic stroke due to acute intracranial ICA occlusion. This approach has allowed us to achieve excellent clinical and angiographic outcomes and shorten the PRT time, and it may reduce the incidence of distal emboli in acute ischemic thrombectomy. This technique should be considered in cases of acute intracranial ICA occlusion when the BGC is blocked by a thrombus during the procedure. However, our findings should be confirmed by future multicenter, large-sample studies.

## Figures and Tables

**Table 1:** Baseline characteristics, clinical features, and preprocedural radiological features of the 32 patients who underwent RTRS.

Item		
Clinical features	Male sex, *n* (%)	12 (37.5%)
	Age, mean ± SD (y)	73 ± 11
	OPT, mean ± SD (min)	382.0 ± 325.9
	TOAST-CE	21 (65.6%)
	TOAST-undetermined: cryptogenic embolism	11 (34.4%)
	Admission NIHSS, IQR	19 (17, 21)

Vascular risk factors	Hypertension, *n* (%)	23 (71.9%)
	Diabetes mellitus, *n* (%)	6 (18.8%)
	Atrial fibrillation, *n* (%)	21 (65.6%)
	Hyperlipidemia, *n* (%)	3 (9.4%)
	History of smoking, *n* (%)	7 (21.9%)

Preprocedural radiological features	CBS, IQR	4.5 (3, 5)

SD: standard deviation; OPT: onset-to-presentation time; TOAST: Trial of Org 10172 in Acute Stroke Treatment; CE: cardioembolism; NIHSS: NIH stroke scale: IQR: interquartile range; CBS: clot burden score.

**Table 2:** Procedure details and radiological and clinical outcomes of the 32 patients who underwent RTRS.

Item		
Procedure detail	One pass of primary clot retrieval, *n* (%)	32 (100%)
	Passes of rescue clot retrieval, IQR	1 (1.3)
	Passes of rescue clot retrieval, median (min, max)	1 (1.8)
	PRT, mean ± SD (min)	59.8 ± 23.1
	PRT, median (min, max) (min)	46 (22.142)

Radiological outcome	One pass of primary clot retrieval achieves	
	≥eTICI 2b reperfusion, *n* (%)	32 (100%)
	≥eTICI 2c reperfusion, *n* (%)	30 (93.8%)
	PPE: eTICI3 reperfusion, *n* (%)	27 (84.4%)
	Distal emboli, *n* (%)	2 (6.3%)
	Emboli to the ACA	0 (0%)
	sICH	0 (0%)

Clinical outcome	Good prognosis	14 (43.8%)
	Mortality	8 (25.0%)

IQR: interquartile range; SD: standard deviation; OPT: onset-to-presentation time; eTICI: Extended treatment in Cerebral Infarction (eTICI) score; ACA: anterior cerebral artery; FPE: first-pass effect; sICH: symptomatic intracranial hemorrhage; PRT: puncture-to-reperfusion time; FPE: first-pass effect.

## Data Availability

We declare that we can make data available on request through institutional review board once it is needed.
